# Delighting Palates with AI: Reinforcement Learning’s Triumph in Crafting Personalized Meal Plans with High User Acceptance

**DOI:** 10.3390/nu16030346

**Published:** 2024-01-24

**Authors:** Maryam Amiri, Fatemeh Sarani Rad, Juan Li

**Affiliations:** Department of Computer Science, North Dakota State University, Fargo, ND 58105, USA; m.amiri@ndsu.edu (M.A.); fatemeh.saranirad@ndsu.edu (F.S.R.)

**Keywords:** meal planning, reinforcement learning, collaborative filtering, multi-criteria decision making, reward shaping

## Abstract

Eating, central to human existence, is influenced by a myriad of factors, including nutrition, health, personal taste, cultural background, and flavor preferences. The challenge of devising personalized meal plans that effectively encompass these dimensions is formidable. A crucial shortfall in many existing meal-planning systems is poor user adherence, often stemming from a disconnect between the plan and the user’s lifestyle, preferences, or unseen eating patterns. Our study introduces a pioneering algorithm, CFRL, which melds reinforcement learning (RL) with collaborative filtering (CF) in a unique synergy. This algorithm not only addresses nutritional and health considerations but also dynamically adapts to and uncovers latent user eating habits, thereby significantly enhancing user acceptance and adherence. CFRL utilizes Markov decision processes (MDPs) for interactive meal recommendations and incorporates a CF-based MDP framework to align with broader user preferences, translated into a shared latent vector space. Central to CFRL is its innovative reward-shaping mechanism, rooted in multi-criteria decision-making that includes user ratings, preferences, and nutritional data. This results in versatile, user-specific meal plans. Our comparative analysis with four baseline methods showcases CFRL’s superior performance in key metrics like user satisfaction and nutritional adequacy. This research underscores the effectiveness of combining RL and CF in personalized meal planning, marking a substantial advancement over traditional approaches.

## 1. Introduction

In the realm of human existence, eating transcends mere sustenance, being deeply influenced by a range of factors, including nutrition, health, personal taste, time constraints, cultural background, and flavor preferences. The complexity of these factors makes the task of creating personalized meal plans both critical and challenging. Traditional meal planning systems, while aiming to cater to nutritional and health needs, often fall short in aligning with the individual’s specific lifestyle, preferences, and unseen eating patterns [1,2,3]. This misalignment frequently leads to poor user adherence, undermining the effectiveness of these systems [1,4]. Personalized meal planning systems are evolving to cater more effectively to individual dietary needs and preferences. Traditional systems often use predefined rules and criteria, creating meal plans based on user profiles. For instance, they might suggest a low-carb meal plan for someone with diabetes or a vegetarian plan for users with specific dietary restrictions [5,6,7,8,9]. Despite their utility, these systems suffer from inflexibility and a lack of adaptability to changing user needs. They are prone to errors and inconsistencies due to conflicting rules, and they struggle to stay updated with the latest dietary knowledge and trends.

To overcome these limitations, some systems have adopted data-driven approaches, like collaborative filtering, to generate meal plans. These systems recommend meals based on the user’s past choices and the preferences of similar users [10,11,12]. However, they too have drawbacks, such as reliance on the quality and quantity of user data, which can be sparse or biased. Moreover, these systems often act as ”black boxes”, offering no explanations for their recommendations, which can affect user trust. They also tend to be reactive, not proactively seeking user feedback, which can lead to irrelevant recommendations.

Building upon the existing body of work in personalized meal planning, recent studies have explored various machine learning techniques to address specific challenges in the field. For example, the authors of [13] employed a range of algorithms, like Gradient Boosting and XG Boost, to predict obesity risk and suggest appropriate meals. This approach signifies a shift towards using predictive analytics in dietary recommendations.

In [14], a case-based approach was introduced, relying on ripple-down rules for diet recommendations. This method represented an early attempt to incorporate rule-based systems in diet planning, focusing on patient attributes and rule actions. A more sophisticated approach was presented in [2], the authors of which developed a knowledge-based recommendation framework. This system used a dual-layer architecture combining qualitative assessments of ingredient suitability with quantitative meal plan optimizations, showcasing an early integration of multifaceted data in meal planning.

In [15], innovative methods, like many-objective optimization and sequence-based deep learning in food recommendation systems, were proposed, aiming for more balanced and robust recommendations.

Additionally, the authors of [16] proposed a hybrid system combining collaborative and content-based filtering for more accurate and diverse recommendations. This was echoed in [17] with a matrix factorization collaborative-based system for restaurant recommendations in Riyadh, comparing various algorithms like NMF, SVD, and SVD++ for enhanced prediction accuracy.

Despite these advancements, there remains a gap in integrating reinforcement learning with collaborative filtering, which is where our work makes its primary contribution. Our approach aims to blend these methodologies to offer dynamic, personalized meal planning, enhancing both the adaptability and effectiveness of dietary recommendations.

Addressing this gap, our study introduces a groundbreaking algorithm, CFRL, which innovatively combines reinforcement learning (RL) with collaborative filtering (CF). This synergy enables CFRL to not only consider nutritional health aspects and other preferences but also dynamically adapt to and reveal latent user eating habits by learning from users’ feedback. This adaptability significantly enhances user acceptance and adherence to the proposed meal plans. CFRL employs Markov decision processes (MDPs) for interactive and responsive meal recommendations, further enriched by a CF-based MDP framework. This framework effectively aligns with broader user preferences, translating them into a shared latent vector space, thus offering a deeper insight into user inclinations.

A key feature of CFRL is its novel reward-shaping mechanism, underpinned by multi-criteria decision-making. This mechanism includes user feedback, preferences, and nutritional data, culminating in versatile, user-specific meal plans. Such a mechanism ensures that the recommendations are not just nutritionally sound but also closely aligned with the user’s personal taste and dietary needs. An innovation of our work lies in the “human-in-the-loop” integration, where the system incorporates continuous user feedback to refine and adapt its meal recommendations. This approach signifies a paradigm shift in personalized meal planning, as it allows for dynamic interactions between the user and the system. The system learns and evolves through this interaction in real time, factoring in the user’s evolving preferences and dietary needs. This dynamic adaptation is a significant leap forward from static meal-planning methodologies, paving the way for a more responsive, personalized, and user-centric dietary planning experience.

Our comprehensive comparative analysis pits CFRL against four other approaches, highlighting its superior performance in critical areas such as user satisfaction, nutritional adequacy, and user acceptance score. These results underscore the efficacy of integrating RL and CF in the domain of personalized meal planning. CFRL marks a significant advancement over conventional approaches, offering a nuanced solution to the complex challenge of personalized meal planning. The study demonstrates the potential of this innovative approach in fostering more intelligent, adaptive, and user-focused dietary planning systems.

The subsequent sections of this paper delve into related work in meal planning (Section 2), detail the CFRL algorithm (Section 3), present the experimental setup and results (Section 4), and provide a summary of our findings and future research directions (Section 5).

## 2. Related Works

The development of personalized meal planning systems has been a subject of considerable interest across various fields, encompassing nutrition science, computer science, and human–computer interaction [1,18,19]. This section reviews the diverse methodologies that have been employed in the quest for effective meal-planning solutions, ranging from rule-based systems to advanced machine learning (ML) techniques.

**Rule-based systems**: Early attempts at automating meal planning predominantly relied on rule-based systems. These systems utilized predefined rules and constraints, often based on nutritional guidelines and dietary requirements, to generate meal plans [5,6,7]. While effective in ensuring basic nutritional adequacy, these systems lacked the flexibility to account for individual preferences or lifestyle changes, often leading to static and repetitive meal recommendations.

**Expert systems:** Closely related to rule-based methods, expert systems leveraged the knowledge of nutritionists and dieticians to create meal plans [8,9]. These systems aimed to mimic human expertise in understanding dietary needs but were limited by the need for constant updates from experts to reflect the latest nutritional research and changing dietary guidelines.

**Machine learning-based approaches**: The integration of ML in meal planning marked a significant shift towards more personalized recommendations. Various ML techniques have been employed, each with its own set of strengths and challenges:

*Collaborative filtering*: Popular in recommendation systems, collaborative filtering algorithms suggest meals based on similar users’ preferences. This method can provide highly personalized recommendations but often struggles with the cold-start problem, where new users or items have insufficient data for accurate predictions [10,11,12].

*Content-based filtering*: This approach recommends meals based on the similarity of their characteristics (e.g., ingredients, nutritional content) to those the user has liked in the past [20]. While effective in maintaining consistency in recommendations, it may lead to a lack of variety in meal plans [21].

*Hybrid systems*: Combining collaborative and content-based filtering, hybrid systems aim to leverage the strengths of both methods to provide more diverse and accurate recommendations [22,23,24,25].

*Neural networks and deep learning*: More recent developments have seen the application of neural networks and deep learning in meal planning [2,26]. These systems can process large datasets and learn complex patterns in user preferences and meal characteristics, offering sophisticated personalization capabilities. For example, researchers proposed a U-HEALTH mobile application [27] that exemplifies the integration of machine learning in meal planning, particularly for diabetic patients. It generates daily meal plans, including three main meals and evening snacks, by leveraging a combination of neural networks and decision trees. The system personalizes meal recommendations based on user-specific factors like health conditions, food preferences, allergies, nutritional content, gender, age, and body metrics calculated through a random forest algorithm. An algorithm proposed in [28] employs Monte-Carlo methods and a Markov decision process to select meals based on constraints such as cost, nutritional profile, and environmental impact. The agent is trained using an ε-greedy policy, effectively generating feasible meal plans without requiring extensive human trial and error.

*Constraint-based systems*: These systems generate meal plans by satisfying a set of user-defined constraints, such as calorie limits, dietary restrictions, or budget [29,30]. While they offer tailored solutions, their effectiveness is highly dependent on the accuracy and comprehensiveness of the constraints provided by the user.

*Evolutionary algorithms*: Some researchers have explored the use of evolutionary algorithms for meal planning [31,32]. These algorithms iteratively evolve meal plans based on user feedback, striving to optimize for factors like nutritional balance, user satisfaction, and variety.

Despite the advancements, personalized meal planning remains challenging due to factors like the diversity of individual dietary needs, changing lifestyle patterns, and the dynamic nature of nutritional science. Ensuring user adherence to meal plans, balancing nutritional adequacy with personal preferences, and handling sparse or incomplete user data are ongoing challenges in this domain. In conclusion, the field of meal planning has seen a wide range of approaches, each contributing valuable insights and methodologies. However, there remains a need for systems that can dynamically adapt to individual user requirements while balancing the multifaceted aspects of nutrition, taste, and personal preferences. The following sections detail our approach to addressing these challenges, presenting the design and implementation of our meal planning system.

## 3. Methodology

### 3.1. System Architecture

**Figure 1:**
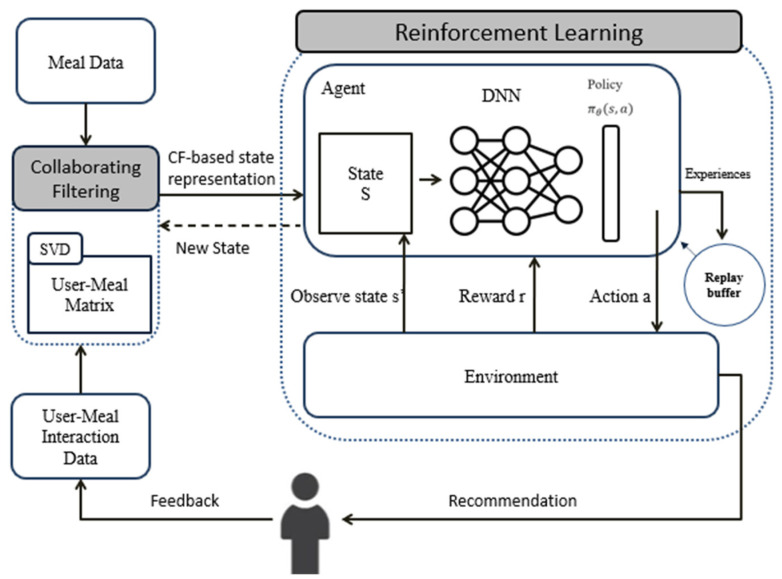
System architecture.

Figure 1 presents the architecture of our proposed methodology, comprising two primary components. The first is a collaborative-filtering-based model designed to predict user acceptance. This model constructs a user–meal matrix, determining user acceptance scores for various meals based on a combination of meal details and user–meal interactions. The meal data encompasses key elements like the name and nutritional information of the meals, while the user–meal interaction data integrate user feedback ratings, which reflect users’ acceptance and preference for each meal.

The second pivotal component is the reinforcement learning (RL) agents. These agents are trained to recommend meals by learning a policy that aligns with the meals’ nutritional value, the users’ health conditions, and their dietary preferences. The RL agents employ a Q-learning algorithm to optimize the expected reward from the suggested meals. This methodology component is crucial as it ensures that the meal suggestions are not only aligned with user preferences but also cater to their specific nutritional and health requirements.

The system architecture can be further explained as follows:

- The user interface is the front-end of the system, where the users can input their personal information, such as age, gender, weight, height, health condition, and dietary restrictions. The user interface also allows the users to rate and provide feedback on the recommended meals, as well as to view their progress and nutritional intake.
- The meal database is the back-end of the system, where the meal data are stored and retrieved. The meal data include the name, ingredients, calories, macronutrients, and micronutrients of each meal. The meal database also stores the user–meal interaction data, such as the ratings and feedback given by the users for each meal.
- The CF component is responsible for predicting the user acceptance scores for each meal based on the meal data and the user–meal interaction data. The CF component uses a matrix factorization technique to learn the latent factors that influence the user meal acceptance. The latent factors are then used to fill the missing entries in the user–meal matrix, resulting in a complete matrix of predicted acceptance scores.
- The RL component is responsible for selecting the best meal to recommend to each user based on the predicted acceptance scores and the user’s health condition. The RL component uses a Q-learning algorithm to learn a policy that maximizes the expected reward from the recommended meals. The reward is defined as a function of the user’s satisfaction and health improvement. The Q-learning algorithm iteratively updates the Q-values of the state–action pairs until they converge to the optimal values.
- The feedback loop is the mechanism that allows the system to learn from the user feedback and improve the recommendations over time. The feedback loop consists of two parts: the user feedback and the system update. The user feedback comprises the rating and the comment given by the user for each recommended meal. The system update is the process of updating the user–meal interaction data and the Q-values based on the user feedback. Users’ feedback is included in both the CF and RL components to allow the system to learn from direct user feedback iteratively.

### 3.2. Acceptance Prediction

Users’ feedback for meal recommendations serves as a direct reflection of individual preferences and acceptance, which are critical in personalizing meal plans. By analyzing the feedback in terms of ratings, we can predict how users might perceive and accept future meal recommendations. This predictive capability is essential for crafting meal plans that resonate with users’ unique taste profiles and dietary requirements. To achieve this, our algorithm utilizes a latent factor model through collaborative filtering, leveraging user ratings to learn low-dimensional embeddings for both users and meals. These embeddings are instrumental in capturing the intricate relationships between users and meals, providing a foundation for generating personalized insights.

We use singular value decomposition (SVD) as a collaborative filtering approach. Singular value decomposition (SVD) is a mathematical approach from linear algebra that has commonly been applied as a method for reducing dimensions in machine learning. SVD serves as a matrix factorization method, with the aim of decreasing the feature count within a dataset by transforming the spatial dimension from an N-dimensional space to a lower K-dimensional space (where K < N). Within the realm of recommender systems, SVD finds utility as a collaborative filtering technique. It employs a matrix structure where individual rows correspond to users and columns correspond to meals. The entries in this matrix signify the ratings users assign to meals [33].

We created a user–meal matrix that maps users to their respective meal ratings and uses it to generate predictions for unrated meals. To optimize meal recommendations based on user rates, our model employs reinforcement learning (RL), a technique that enables an agent to learn how to achieve its goals through trial and error. Specifically, we use a deep q-network, an algorithm that uses a neural network to approximate the expected future reward for carrying out an action in a given state [34].

The state is defined as a comprehensive vector embodying the user’s dietary preferences and various attributes of meals, enriched with details such as cuisine type, ingredients, nutritional content, and the user’s historical meal interactions. For instance, a pronounced user inclination towards Italian cuisine or low-carb meals would be reflected through heightened values in the corresponding elements of the state vector.

The collaborative filtering model in our system is conceptualized through the equation:

$$R=U\times M^{T}$$

where *R* represents the user–meal interaction matrix, this matrix being constructed based on the ratings or feedback provided by users, encapsulating their acceptance and preferences for various meals. Each element *R_ij_* in this matrix signifies the rating given by user *i* to meal *j*, illustrating their level of preference or aversion. The matrix *U* denotes the user embedding matrix, where each row is a latent feature vector representing the tastes and preferences of an individual user. Similarly, *M* symbolizes the meal embedding matrix, with each row being a latent feature vector encapsulating the characteristics of a particular meal. These latent feature vectors are learned through matrix factorization techniques, such as SVD or NMF, which decompose the user–meal interaction matrix into these lower-dimensional, dense representations. This decomposition allows for capturing the underlying patterns in user preferences and meal attributes, enabling the system to make sophisticated and personalized meal recommendations. The collaborative filtering approach, therefore, hinges on the interplay of these latent factors, leveraging the collective preferences and feedback of the user community to recommend meals that align with an individual user’s unique dietary profile and preferences.

### 3.3. Reinforcement Learning-Based Adapted Meal Plan

In our CFRL system, the reinforcement learning (RL) agents are pivotal in optimizing the recommendations for personalized meal plans. These agents employ a Q-learning algorithm [35,36], a type of model-free reinforcement learning, to maximize the expected reward from each meal suggestion. The underlying mechanism involves updating a comprehensive state vector, which encompasses details such as cuisine type, ingredients, nutritional content, and the user’s historical meal interactions. This state vector forms the basis for generating tailored meal recommendations.

The RL component of our system operates with a deep q-network (DQN) algorithm. The DQN, an advanced RL approach, combines traditional Q-learning with deep neural networks. This combination allows the system to process complex input states and learn optimal actions for a vast number of possible meal combinations. The state vector in the DQN is enriched with multidimensional data representing user dietary preferences and meal attributes, making the recommendations highly personalized and context-aware.

In our Q-learning algorithm, we used the following parameters and values:

Learning rate (lr): This is the parameter that controls how much the Q-values are updated based on the observed reward and the next state. We set the learning rate to 0.01, which is a common value for this parameter.

Discount factor (gamma): This is the parameter that controls how much the agent values future rewards compared to immediate rewards. We set the discount factor to 0.95, which is a common value for this parameter.

Exploration rate (epsilon): This is the parameter that controls how much the agent explores new actions versus exploiting known actions. We set the exploration rate to 1.0, which means that the agent starts with a high level of exploration and then gradually reduces it over time.

Neural network architecture: We used a feed-forward neural network with three hidden layers of 128, 64, and 32 units, respectively, and ReLU activation functions. The output layer had a linear activation function, and the loss function was mean-squared error. The optimizer was Adam with a learning rate of 0.01.

We chose the values for the first three parameters based on a literature review and empirical testing [37,38]. We found that these values provided a good balance between exploration and exploitation and resulted in a high-quality policy for our problem and data.

The CFRL approach integrates collaborative filtering (CF) and reinforcement learning (RL) to create a robust selection system for interactive recommendations. The CF component encodes the states of all users into a shared latent vector space using matrix factorization, while the RL component formulates the interactive recommendation problem as an agent-environment RL task and employs a Q-network learning method based on deep Q-learning (DQN). The integration involves the development of a novel CF-based MDP, where the states of all users are used to define the state space of the MDP. This collaborative state representation enables the system to make recommendations that reflect the collective preferences of the user community. By learning collaborative recommendations that adapt to individual user feedback while leveraging the collective preferences of the user community, the CFRL approach provides a robust and interactive recommendation system over multiple rounds.

#### 3.3.1. Reward Shaping and Optimization

Central to our RL approach is the sophisticated reward function, which plays a crucial role in guiding the learning process of the RL agents. This reward function is an intricate blend of user feedback, preference factors, satisfaction, and health indices. It is mathematically formulated as a weighted sum of three components: user rating ($r_{u}$), nutrition score (*n*), and preference score (p). These are represented as:

(1) $$r=w_{r}r_{u}+w_{n}n+w_{p}p$$

In this equation, weights $w_{r}$, $w_{n}$, and $w_{p}$ denote the importance of each component, calibrated through multi-criteria decision-making. The user rating ($r_{u}$) reflects the user’s feedback on the meal. Ratings range from 1 to 5 and are normalized to 0 to 1. The nutrition score (*n*) is assessed using the prerow value (*PV*) [39,40], which evaluates the nutritional balance of the meal. The *PV* is calculated as:

(2) $$PV=min\left[\mu \left(x_{i}\right)\right]\cdot \left(n-1\right)\cdot {\left(\sum_{i\neq i_{min}}\frac{1}{\mu \left(x_{i}\right)}\right)}^{-1}$$

In this formula, *μ(x_i_)* represents the membership value of each nutrient in the meal, and n is the number of observed nutrients. The preference score (*p*) is derived from a multi-objective optimization algorithm, combining the Technique for Order of Preference by Similarity to Ideal Solution (TOPSIS) [41] and Analytic Hierarchy Process (AHP) [42,43] methods. This algorithm evaluates meals based on multiple criteria, like nutrition, taste, cost, and convenience. It involves a process of defining criteria, weighing each criterion using the AHP, normalizing and weighting the criteria values, determining ideal solutions, calculating distances, and sorting meals based on their closeness to the ideal solution.

The algorithm is described in detail in the following steps:

1. Define the criteria for choosing the best meals, such as nutrition, taste, cost, and convenience.
2. Determine the weighting of each criterion based on the user’s preferences using the AHP method: creating a pairwise comparison matrix N × N using the weighting score and establishing a performance decision matrix $A_{ij}={\left(a_{ij}\right)}_{m\times n}$ consisting of m meals and n different preferences:
  (3) $$A_{ij}=\left[\begin{array}{ccc} a_{11} & \cdots & a_{1n}\\ \vdots & \ddots & \vdots \\ a_{m1} & \cdots & a_{mn} \end{array}\right]$$
3. Normalize the criteria values to make sure that each criterion has the same impact on the final decision. Normalize the $A_{ij}$ matrix to the matrix $R={\left(r_{ij}\right)}_{m\times n}$
  (4) $$r_{ij}=\frac{x_{ij}}{\sqrt{\sum_{k=1}^{m}x_{kj}^{2}}},i=1,2,\cdots ,mj=1,2,\cdots ,n$$
4. Calculate the weighted normalization of each meal based on the criteria values and the weights determined in step two.
  (5) $$t_{ij}=r_{ij}\cdot w_{j},i=1,2,\cdots ,mj=1,2,\cdots ,n$$
  where $w_{j}=W_{j}/\sum_{k=1}^{n}W_{k}$, j=1,2,⋯,n, so that $\sum_{i=1}^{n}{\;w}_{i}=1$ and $W_{j}$ is the original weight given to the indicator $v_{j}$, j=1,2,⋯,n.
5. Calculate the best (positive ideal) solution and the worst (negative ideal) solution based on the weighted normalization values:
  $A_{w}=\left\{\left\langle max\left(t_{ij}|i=1,2,\cdots ,m\right)|j\in J_{-}\right\rangle ,\left\langle min\left(t_{ij}|i=1,2,\cdots ,m\right)|j\in J_{+}\right\rangle \right\}\equiv \left\{t_{wj}|j=1,2,\cdots ,n\right\}$,
  $A_{b}=\left\{\left\langle min\left(t_{ij}|i=1,2,\cdots ,m\right)|j\in J_{-}\right\rangle ,\left\langle max\left(t_{ij}|i=1,2,\cdots ,m\right)|j\in J_{+}\right\rangle \right\}\equiv \left\{t_{bj}|j=1,2,\cdots ,n\right\}$ where $J_{+}=\left\{j=1,2,\cdots ,n|j\right\}$ is associated with the criteria having a positive impact, and
  $J_{-}=\left\{j=1,2,\cdots ,n|j\right\}$ is associated with the criteria having a negative impact.
6. Calculate the Euclidean distance between each meal and the best and worst solutions.
  The distance between the target alternative i and the worst condition $A_{w}$ is as follows:
  $$d_{iw}=\sqrt{\sum_{j=1}^{n}{\left(t_{ij}-t_{wj}\right)}^{2}},\;i=1,2,\cdots ,m$$
  and the distance between the alternative i and the best condition $A_{b}$ is as follows:
  $$d_{ib}=\sqrt{\sum_{j=1}^{n}{\left(t_{ij}-t_{bj}\right)}^{2}},\;i=1,2,\cdots ,m$$
  where $d_{iw}$ and $d_{ib}$ are Euclidean distances from the target alternative i to the worst and best conditions, respectively.
7. Calculate the meal’s similarity to the worst alternative (The higher TOPSIS score, the better).
  $$S_{i}=\frac{d_{iw}}{d_{iw}+d_{ib}},i=1,2,\cdots ,m$$
8. Sort the meals based on the TOPSIS score. The meal with the highest TOPSIS score is the best option according to the user’s preferences.

Our RL training framework is grounded in an actor–critic architecture. This architecture comprises two primary components: the actor, which proposes actions (meal recommendations), and the critic, which evaluates these actions based on the reward function. The actor–critic method provides a balanced approach to learning, allowing for more stable and efficient convergence in training the RL agents. In Algorithm 1, we present the proposed algorithm, which outlines the key steps and procedures involved in our approach. This pseudocode provides a detailed overview of the algorithm’s structure and operational flow.
**Algorithm 1.** CFRL algorithm. Algorithm: Personalized Adaptive Meal Recommendation (CFRL) Input: - U_train: User latent features after MF on user–item interaction matrix; - R: User–item rating matrix; - K: Number of episodes for training; - T: Number of time steps per episode; - γ (gamma): Discount factor for future rewards; - ε (epsilon): Exploration rate for ε-greedy strategy; - M: Memory buffer to store transitions.  Output: - Q^^^: Learned Q-network for action-value estimation; - all_results_df: DataFrame containing the results from all experiments.Procedure: Initialize recommender with user–item interaction matrix R and item feature matrix V. For each combination of learning rate α and discount factor γ:    Initialize Q^^^ with random weights.    For each episode k from 1 to K:        Pick a user u from U_train as the environment.        Observe initial raw state *s*0.        Compute CF-based state *st* using user latent features U_u.        For each time step t from 0 to T-1:            Select action *at* using ε-greedy policy with respect to Q^^^.            Take action *at*, observe reward *rt* + 1 and raw state *s′t* + 1.            Take CF-based state *st* + 1 by updating U_u based on the latest rating *Rui*.            Store transition (*st*,*at*,*rt* + 1,*st* + 1) in M.            Sample a minibatch from M and update Q^^^′s weights w according to:               *w*←*w* + *α*[*r* + *γ*max*aQ^^^*(*st* + 1,*a*)−*Q*^(*st*,*at*,*w*)]∇*wQ^^^*(*st*,*at*,*w*)        Append episode results to the results list. Combine all episode results into a DataFrame. Return the all_results_df. Note: the reward *r* is computed based on Equation (1).

#### 3.3.2. Human-in-the-Loop Integration

A critical aspect of our CFRL system is the integration of the ‘human-in-the-loop’ methodology, which significantly influences both the reinforcement learning and collaborative filtering components of the system. This approach ensures that the system remains responsive and adaptive to individual user feedback, playing a central role in continually refining the meal recommendation process.

Within the reinforcement learning framework, the human-in-the-loop approach is fundamental. It allows the system to iteratively learn and adapt from direct user feedback. Each user interaction, particularly their ratings and satisfaction with recommended meals, serves as a vital reward signal. This signal is a composite reflection of user satisfaction, nutritional value, and other influencing factors. Utilizing this feedback, the reinforcement learning agent updates the state vector, which is enriched with a user’s dietary inclinations and specific meal attributes. The updated state vector becomes the foundation for generating subsequent personalized meal recommendations, ensuring that each recommendation is tailored to the individual’s tastes and aligned with their nutritional and health objectives.

The proposed approach is an interactive process where the recommender system gives an item to the user, gets a rating from the user, and updates its user knowledge based on the rating. This interaction lets the system adjust its recommendations over time. The user feedback shapes the recommendations by updating the user feature, which captures the user’s changing preferences. The user feedback also helps the system treat the recommendation problem as a reinforcement learning (RL) task, where the system learns from the user’s feedback to optimize its policy and make better recommendations. The system improves its recommendations based on the rewards (ratings) from the user. The user feedback at each stage helps the system adapt to individual user preferences, learn from the ratings, and make collaborative recommendations that match the collective user preferences, improving the recommendations over time.

In the realm of collaborative filtering, human-in-the-loop integration is crucial for capturing and understanding the nuanced preferences of users. The system dynamically adjusts the user–meal matrix based on continuous user ratings and feedback. This ongoing influx of user-specific data allows the collaborative filtering model to evolve and improve its prediction accuracy. As a result, the meal recommendations become increasingly aligned with the user’s personal taste and nutritional needs.

The synergy between reinforcement learning and collaborative filtering, enriched by real-time user feedback, forms the backbone of the CFRL system. By defining a state vector that encapsulates a user’s dietary inclinations and specific meal attributes and employing a reinforcement learning algorithm, our approach presents a comprehensive, adaptable, and user-centric approach to personalized meal recommendations. The human-in-the-loop integration thus ensures a dynamic, responsive, and evolving meal-planning system capable of delivering highly personalized and satisfactory meal-planning solutions.

## 4. Results

The primary objective of the evaluation phase in our study was to thoroughly assess and understand the performance of our proposed CFRL algorithm in the context of personalized meal planning. To achieve this, we employed a dual approach in our experiments, comprising both use case studies and quantitative analysis, to compare our methodology with existing meal-planning approaches.

Our first approach involved use case studies, where we created imaginary user profiles with specific dietary preferences and requirements. These use cases allowed us to test the adaptability and effectiveness of our meal-planning algorithm in real-world scenarios. By simulating various user types, each with unique dietary needs and preferences, we could evaluate how well the CFRL system responds to diverse user profiles. This approach provided us with insights into the practical applicability of our system and its capacity to generate personalized meal plans that cater to individual user needs.

The second part of our evaluation was a quantitative study where we compared our CFRL approach with other existing meal-planning methods. The criteria for comparison included the ability of each method to provide nutritionally balanced meal plans, adherence to user preferences, and the likelihood of user acceptance of the recommended meals. For this quantitative analysis, we utilized a real dataset comprising various recipes and user ratings of these meals. This dataset served as a rich source of empirical data, allowing us to objectively measure and compare the performance of our algorithm against established benchmarks in the field.

Our preprocessing involved two main datasets: one containing over 180,000 recipes and another with more than 700,000 user interactions, including reviews, from Food.com, spanning 18 years (2000–2018). To ensure the relevance and reliability of our data, we only included recipes that had received at least 10 feedback interactions from distinct users. This filtering helped us focus on recipes with sufficient user engagement for meaningful analysis.

We implemented our meal-planning algorithm, incorporating the CF-based state representation and the reinforcement learning model. For comparative analysis, we also implemented traditional static meal-planning methods that are commonly used in the field.

The dataset was randomly partitioned into training (70%) and testing sets (30%) for a balanced evaluation. The training set was used to educate and fine-tune our algorithm, while the testing set served for assessment purposes. We performed 10 iterations of the experiments to account for any potential variations in the results.

The evaluation of the proposed algorithm focused on the following metrics:

- Acceptance score;
- Preference score;
- Adherence to dietary goals—PV.

Using the iterative grid search method, we exhaustively explored a predefined range of values for each parameter to fine-tune the parameters of a model or algorithm. Various parameter combinations were generated, and the model’s performance was evaluated for each combination. Model parameters were adjusted, evaluated, and refined iteratively based on evaluation results in an iterative process. This method systematically explores a model’s parameters to find the best settings that optimize its performance. Using this approach, parameter tuning becomes more accessible and more data-driven. In the case of many parameters or large parameter spaces, it can be computationally expensive. In such cases, Bayesian optimization can be used to improve its efficiency [44]. Our model was trained for 300 episodes, and we used a discount factor of 0.99 and a learning rate of 25 × 10^−4^ for the reinforcement learning algorithm. PV, acceptance, and preference scores had weights of 0.4, 0.3, and 0.3, respectively, in the reward function.

### 4.1. Use Case Study

The development of a robust meal recommendation system hinges on its capacity to adapt to individual user preferences, dietary restrictions, and nutritional needs while also incorporating feedback to refine its suggestions over time. This iterative process, akin to a conversation between the user and the system, leverages the principles of reinforcement learning (RL) to achieve a harmonious balance between the exploration of new meal options and the exploitation of known user preferences.

#### 4.1.1. Initial Episode

In this particular use case, our system addressed the needs of a 45-year-old female with distinct dietary requirements and taste preferences. Her diet was constrained by gluten intolerance and a need for low-sodium options, and she had a specific liking for American cuisine. To cater to these requirements, the system’s initial recommendations were carefully curated to align with her health and dietary restrictions while also considering her calorie intake and budget limitations. The effectiveness of these recommendations was assessed using two metrics: the prerow value (PV) and the preference score. The PV metric measures how well the recommended meals match the user’s health condition and dietary restrictions. A PV score of 0.7 or higher indicates that the recommended meals are acceptable for the user, while a PV score of 0.9 or higher indicates that the recommended meals are optimal for the user. The preference score measures how much the user likes the recommended meals based on their taste and satisfaction. A preference score of 0.8 or higher indicates that the user is highly satisfied with the recommended meals. User acceptance was then gauged on a scale from 1 to 5, where 1 means very dissatisfied and 5 means very satisfied. User acceptance provides a comprehensive measure of the system’s performance in meeting the user’s needs.

Table 1 shows the recommended meals for the use case in the first episode, along with their nutrition, preference scores, PVs, and acceptance scores. The table columns are as follows:

- Meal type: the type of meal, such as breakfast, lunch, or dinner.
- Meal name: the name of the meal, such as country-style breakfast potatoes, chile rellenos, or grilled shrimp with garlic and herbs.
- Nutrition: the nutritional information for the meal, such as calories, carbohydrates, sodium, sugar, fiber, protein, and fat.
- Preference score [0–1]: the preference score of the user for the meal, ranging from 0 to 1, where 0 means very dissatisfied and 1 means very satisfied.
- PV: the prerow value of the meal, ranging from 0 to 1, where 0 means very unhealthy and 1 means very healthy.
- Acceptance score [1–5]: the acceptance score of the user for the meal, ranging from 1 to 5, where 1 means very dissatisfied and 5 means very satisfied.

**Table 1:** Recommended meal for the use case in the first episode.

Meal Type	Meal Name	Nutrition	Preference Score [0–1]	PV	Acceptance Score [1–5]
Breakfast	Country-style breakfast potatoes	Calories: 343, carbohydrates: 37 g, sodium: 39 mg, sugar: 4.2 g, fiber: 4.2 g, protein: 4.4 g, fat: 20 g	0.80	0.89	3
Lunch	Chile rellenos	Calories: 510, carbohydrates: 27.5 g, sodium: 900 mg, sugar: 16 g, fiber: 10 g, protein: 33.5 g, fat: 32 g	0.89	0.73	3
Dinner	Grilled shrimp with garlic and herbs	Calories: 240, carbohydrates: 7 g, sodium: 1280 mg, sugar: 3.5 g, fiber: 1 g, protein: 31 g, fat: 9 g	0.73	0.89	5

As illustrated in the table, the meals suggested were varied, aligning with the user’s health guidelines but with room for refinement in terms of personal taste and acceptance scores. For example, the breakfast meal had a high PV score but a low acceptance score, indicating that the user did not like the taste of the meal. The lunch meal had a high preference score but a low PV score, indicating that the user liked the taste of the meal, but it was not very healthy for them. The dinner meal had a high PV score and a high acceptance score, indicating that the user liked the taste of the meal, and it was also healthy for them. These results demonstrate the trade-off between the user’s preferences and health conditions and the need for the system to balance them in the recommendations.

#### 4.1.2. Iterative Improvement

Over the course of 150 episodes, the algorithm demonstrated its adaptive nature. As illustrated in Table 2, each meal’s performance was evaluated using a composite metric encompassing preference, nutrition (PV), and acceptance scores. The RL component of the system processed the user’s feedback—implicit or explicit—adjusting the recommendation policy to better align with the user’s unique tastes and nutritional objectives. This feedback loop is pivotal; it informs the system not only about the user’s satisfaction with the meals but also about the nuanced interplay of flavors, ingredients, and preparation styles that resonate with the user.

The convergence we observed in the recommendation quality over successive episodes is a testament to the efficacy of collaborative filtering (CF) in conjunction with RL. CF contributes by drawing on the collective experiences of similar users to predict preferences, which, when combined with the RL agent’s policy, hones the recommendations to the user’s evolving palate. The gradual increase in preference and PV scores, coupled with consistently high acceptance rates, underscores the system’s ability to learn and adapt.

By the 150th episode, the system had discernibly improved, with preference scores reaching their zenith, indicating a high level of user satisfaction. PV scores remained robust, ensuring that nutritional quality was not compromised for taste alone. The acceptance scores were also strong, showing that the meals suggested were well received and likely to be embraced in the user’s dietary routine.

The culminating meal plan presented in Table 3 reflects the system’s learning journey—it is the product of a carefully calibrated algorithm that considers health constraints, cost, and cooking time, all through the lens of the user’s dietary journey. The high PV score of 0.98549 is indicative of a nutritionally balanced meal plan that does not just meet but exceeds the user’s health requirements. This, paired with the high preference and acceptance scores, signifies a well-rounded meal plan that the user is likely to enjoy and adopt.

### 4.2. Qualitative Study

To evaluate our framework (Adaptive Meal Recommender) feasibility, we compared it with the following methods:

- CF-based method [45]: the method uses collaborative filtering for meal recommendations.
- Nutrition-based method [46]: reinforcement learning method in which the reward function is based on a combination of nutrition score and preference score.
- Nutrition- and preference-based method [47]: the nutrition score is used for the cost function, and the preference score is used as the TOPSIS method criteria.

#### Performance Metrics Results

The experiments were conducted on datasets with mean values of 10 runs for each method with 1000 users. The results showed that our algorithm outperformed all the baselines on most metrics, demonstrating its effectiveness and efficiency for meal planning.

The primary metric that we used to measure the performance of our algorithm was the acceptance score, which reflects how satisfied the users were with the generated meal plans. The acceptance score was calculated as the average rating given by the users to the meal plans on a scale of 1 to 5, where 1 means very dissatisfied and 5 means very satisfied. Our algorithm achieved the highest acceptance score of 4.5, significantly higher than nutrition-based and nutrition- and preference-based methods (*p*-value < 0.001, ANOVA test), indicating that it generated meal plans that were highly satisfying for the users. This result shows that our algorithm successfully learned from the user rating and adapted the meal plans accordingly.

Another metric that we used to measure the performance of our algorithm was the preference score, which reflects how well the meal plans matched the users’ preferences. The preference score was calculated as the average score obtained by TOPSIS on a scale of 0 to 1, where 0 means no similarity and 1 means perfect similarity. Our algorithm achieved a high preference score of 0.84, which was significantly higher than the CF-based method (0.6, *p*-value < 0.0001), but lower than the nutrition- and preference-based method (0.86) and the fuzzy-based method (0.92). This result shows that our algorithm balanced the trade-off between preference and nutrition as it did not generate meal plans that were only preferable but unhealthy or healthy but undesirable.

The last metric we used to measure the performance of our algorithm was the PV score, which reflects how well the meal plans met the nutritional requirements of the users. The PV score was calculated based on the prerow value equation, which was based on the nutritional values (such as calories, protein, fat, etc.) that were within the recommended range for each user on a scale of 0 to 1, where 0 means none of the values were within the range and 1 means all of them were within the range. Our algorithm achieved a high PV score of 0.9, which was approximately equal to the nutrition- and preference-based method and the fuzzy-based method (0.85) but significantly higher than the CF-based method (0.42, *p*-value < 0.0001). This result shows that our algorithm did not compromise the nutritional quality of the meal plans, as it generated meal plans consistent with the user’s dietary goals and needs. The results are presented in Table 4.

## 5. Conclusions and Future Work

In this paper, we proposed a novel algorithm for meal planning that combines reinforcement learning (RL), collaborative filtering (CF), and reward-shaping techniques. We evaluated our algorithm using real-world user data and compared it with several baselines, including traditional static methods. The results suggested that our algorithm performed better than the baselines on most metrics, indicating its potential for meal planning.

The main contribution of our work is that we integrated RL and CF techniques in a unified framework that leverages the strengths of each method. By shaping rewards using user ratings and considering nutritional information, our algorithm-generated meal plans are tailored to individual tastes and dietary requirements. Moreover, using RL to learn and adapt meal plans based on user feedback and preferences, our algorithm provides flexibility, adaptability, and personalization.

However, our work also has some limitations and potential biases that need to be acknowledged and addressed. First, our data sources may suffer from selection bias, as they may not represent the diversity of users and their preferences. Second, our algorithm design may reinforce existing user preferences, as it relies on user feedback and collaborative filtering, which may overlook novel but relevant meal suggestions. Third, our algorithm may not generalize well to the broader population, as it depends on user feedback, which may vary across different groups and contexts.

In future work, we aim to further improve the performance and capabilities of our algorithm by considering additional factors and mitigating the biases and constraints. Some possible directions for future research are:

- Incorporating temporal dynamics into the meal planning process. The algorithm can provide more contextually relevant and diverse meal plans by considering time-dependent factors such as daily routines, seasonal variations, or specific occasions.
- Incorporating ingredient availability into meal planning. By considering the availability of ingredients in users’ local areas or adjusting the meal plans based on ingredient substitutions, the algorithm can offer practical and feasible meal options.
- Integrating other machine learning techniques and data sources to enhance the accuracy and personalization of our algorithm. Hybrid approaches that combine RL and CF techniques with different machine learning algorithms and data sources may provide even better recommendations and adaptability to users’ changing preferences and needs. For example, we can use deep learning to learn more complex and nonlinear patterns from the data or use multi-agent systems to model the interactions and cooperation among users.
- Designing a user interface to facilitate user interaction and feedback. The algorithm can benefit from a user-friendly and intuitive interface that allows users to easily select, rate, and modify their meal plans, as well as provide suggestions and comments. The interface can also display the nutritional information, user ratings, and other relevant data for each meal option, as well as the overall progress and performance of the algorithm.
- Analyzing the results and impacts of our algorithm on user behavior and health outcomes. The algorithm can be evaluated using various metrics and methods, such as user satisfaction, adherence, retention, diversity, novelty, and serendipity. The algorithm can also be tested in different scenarios and settings, such as long-term studies, randomized controlled trials, or real-world deployments. The algorithm can also be compared with other existing or emerging meal planning systems, as well as human experts or nutritionists.

Our algorithm holds great promise in providing personalized and adaptable meal plans that promote user satisfaction and adherence to dietary goals. Continued research and development in this area will improve the effectiveness and practicality of meal planning systems, ultimately promoting healthier and more enjoyable eating habits.

## Figures and Tables

**Table 2:** Recommended meal for the use case in the 150th episode.

Meal Type	Meal Name	Nutrition	Preference Score [0–1]	PV	Acceptance Score [1–5]
Breakfast	Oatmeal waffles or pancakes	Calories: 105, carbohydrates: 17 g, sodium: 351 mg, sugar: 1.3 g, fiber: 1.8 g, protein: 3.4 g, fat: 2.5 g	1	0.83	4
Lunch	Fourth of July grilled salmon or chicken	Calories: 199, carbohydrates: 4.6 g, sodium: 660 mg, sugar: 3.4 g, fiber: 1 g, protein: 6.2 g, fat: 17 g	0.89	0.87	5
Dinner	Amazon fried chicken breasts with cilantro sauce	Calories: 791, carbohydrates: 46 g, sodium: 919 mg, sugar: 5.8 g, fiber: 2.5 g, protein: 47 g, fat: 45 g	0.88	0.76	5

**Table 3:** Recommended meal for the use case in the last episode.

Meal Type	Meal Name	Nutrition	Preference Score [0–1]	PV	Acceptance Score [1–5]
Breakfast	Scrambled eggs	Calories: 216.6, carbohydrates: 2.6 g, sodium: 578 mg, sugar: 0.7 g, fiber: 0 g, protein: 11.9 g, fat: 17.4 g	0.92	0.81	5
Lunch	Grilled margarita chicken breasts	Calories: 316.4, carbohydrates: 1.3 g, sodium: 506 mg, sugar: 0.3 g, fiber: 0.1 g, protein: 29.3 g, fat: 21.1 g	0.94	0.95	5
Dinner	Fattoush salad	Calories: 297.9, carbohydrates: 38.1 g, sodium: 240 mg, sugar: 8.3 g, fiber: 4.6 g, protein: 6.6 g, fat: 14.7 g	0.88	1	5

**Table 4:** Performance comparison of different methods.

Method	Avg PV [0–1]	Avg Preference Score [0–1]	Avg Acceptance Score [1–5]
**CFRL**	**0.9**	**0.84**	**4.5**
CF-based	0.42	0.6	4.5
Nutrition-based	0.94	0.86	2.8
Nutrition- and preference-based	0.85	0.92	3.8

The bold numbers show the proposed algorithm performance.

## Data Availability

Data was obtained from Kaggle.com and are available at https://www.kaggle.com/datasets/shuyangli94/food-com-recipes-and-user-interactions (accessed on 22 December 2023).
